# Chronic Ocular Surface Pain: A Missing Member of the Chronic Overlapping Pain Conditions?

**DOI:** 10.1007/s40265-026-02343-9

**Published:** 2026-06-10

**Authors:** Jonghoon Chang, Alex Hattenhauer, Luisa Saccaro, Ferida Mamatkazina, Elizabeth Felix, Lindsey De Lott, Anat Galor

**Affiliations:** 1 Bascom Palmer Eye Institute, University of Miami Miller School of Medicine, Miami, FL USA; 2 Surgical Services, Miami Veterans Affairs Medical Center, Miami, FL USA; 3 Bogomolets National Medical University, Kyiv, Ukraine; 4 Research Services, Miami Veterans Affairs Medical Center, Miami, FL USA; 5 Department of Physical Medicine and Rehabilitation, University of Miami Miller School of Medicine, Miami, FL USA; 6 Department of Ophthalmology and Visual Sciences, University of Michigan, Ann Arbor, MI USA

## Abstract

Chronic overlapping pain conditions (COPCs) comprise a cluster of ten chronic pain disorders that frequently co-occur and are conceptualized as sharing centrally mediated or nociplastic mechanisms. Chronic ocular surface pain (COSP), defined as ocular surface pain lasting more than three months, has traditionally been classified under “dry eye disease.” However, emerging evidence suggests that in a subset of individuals, COSP shares important clinical and mechanistic features with COPCs, supporting the need for an updated synthesis as increasing data implicate central contributions to pain. This narrative review examines COSP within the broader COPC framework across epidemiology, risk factors, pathophysiology, diagnosis, and treatment, and proposes that in select cases, COSP may warrant conceptual consideration as an additional COPC. Across the literature, COSP shares several similarities with COPCs, including high prevalence, female predominance, and increasing frequency with age. Chronic ocular surface pain also commonly clusters with COPCs and, when nociplastic features are present, is associated with psychosocial comorbidity. Mechanistically, COSP in some individuals demonstrates characteristics of nociplastic pain, including symptom–sign discordance, persistent pain despite topical anesthesia, multisite hyperalgesia, and altered functional connectivity within central pain and sensory processing networks. Collectively, current evidence supports substantial overlap between COSP and COPCs within a nociplastic framework, suggesting that COSP may be best understood, in part, within this broader construct. Recognizing COSP in the context of COPCs has important implications for mechanism-based approaches to diagnosis, treatment, and future research aimed at improving outcomes.

## Key Points

**Table:** 

Review chronic ocular surface pain (COSP) in the context of current literature and reconsider “Dry Eye Disease” from a pain-centered perspective.	
Evaluate COSP within the framework of chronic overlapping pain conditions (COPCs), highlighting shared epidemiology, risk factors, pathophysiology, clinical features and treatment, and assess its potential classification as an eleventh COPC.	

## Introduction

One in five adults in the USA reports chronic pain, defined by the International Association for the Study of Pain (IASP) as "an unpleasant sensory and emotional experience associated with, or resembling that associated with, actual or potential tissue damage"[1] lasting longer than three months [2]. Chronic overlapping pain conditions (COPCs) refer to a cluster of ten disorders that frequently co-occur at higher rates than expected given the prevalence in the general population [2, 3]. These conditions include chronic low back pain (cLBP), chronic migraine, irritable bowel syndrome (IBS), temporomandibular disorder (TMD), chronic tension-type headache (cTTH), interstitial cystitis/bladder pain syndrome (IC/BPS), fibromyalgia (FM), myalgic encephalomyelitis/chronic fatigue syndrome (ME/CFS), vulvodynia, and endometriosis [2, 3]. While COPCs can present with or without identifiable tissue damage [4, 5], nociplastic pain (or pain that arises from altered nociceptive processing without clear evidence of actual tissue damage or disease in the somatosensory system [6, 7]) serves as the potential unifying mechanism underlying their co-occurrence [4, 8].

Chronic ocular surface pain (COSP) shares many characteristics with COPCs. Chronic ocular surface pain is defined as pain or discomfort experienced at the ocular surface (cornea and conjunctiva) lasting more than three months [9]. Although COSP may be initiated by various triggers (e.g., inflammation, infection, or trauma [10, 11]), clinical nociplastic features such as the presence of multisite pain and heightened sensory awareness are frequently underrecognized [12].

**Fig. 1:**
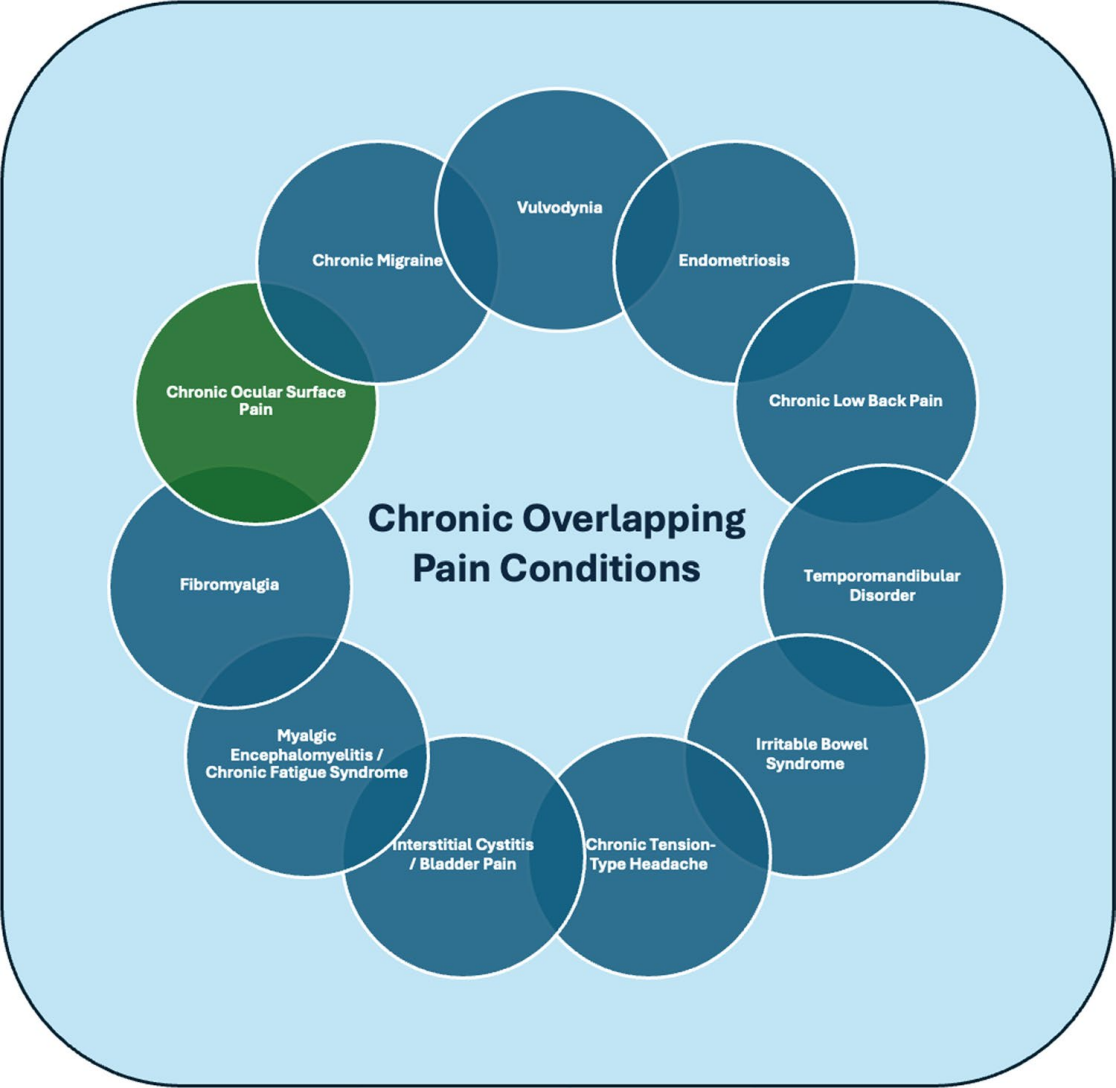
The ten recognized chronic overlapping pain conditions (COPCs), with chronic ocular surface pain (COSP) proposed as an additional, eleventh condition for consideration

This narrative review explores COSP within the broader framework of COPCs (Fig. 1), examining similarities and differences in epidemiology, risk factors, pathophysiology, diagnosis, and treatment. Special emphasis is placed on recognizing nociplastic features among individuals with COSP and implementing a multifaceted clinical approach to treatment by drawing on current knowledge of COPCs.

### Literature Selection, Definitions and Current Limitation on Terminology

The literature review was conducted using the search terms “dry eye,” “dry eye syndrome,” “dry eye symptoms,” and “dry eye disease,” in combination with “chronic ocular surface pain,” “ocular surface pain,” “ocular pain,” “neuropathic ocular/corneal pain,” and “nociplastic ocular pain.” This approach was employed because much of the literature relevant to COSP is embedded within the broader dry eye disease framework. This highlights an important limitation of the field, as symptoms attributed to COSP may arise from dry eye disease or from other distinct mechanisms that were not consistently differentiated in prior studies. For COPCs, both individual condition names and the term “COPCs” were used in the literature search. Definitions of key pain-related terminology are provided in Table 1.

**Table 1:** Key pain terminology and definitions

Term	Definition
OSP (ocular surface pain) [10]	Pain localized to the ocular surface (without a specified duration or temporal criterion)
COSP (chronic ocular surface pain) [9]	Pain localized to the ocular surface that persists for at least 3 months, aligning with definitions of chronic pain
NCP (neuropathic corneal pain) [135]	A subtype of ocular surface pain resulting from a lesion or disease affecting peripheral corneal nerves (or other ocular surface nerves). This may arise from systemic conditions that damage nerves (e.g., Sjögren’s syndrome, diabetes mellitus) or from local trauma (e.g., surgery)
DED (dry eye disease) [180]	A heterogeneous and variably defined term that has been used to describe both symptoms (including pain) and signs of ocular surface and tear film abnormalities (e.g., decreased tear production, tear instability, epithelial damage). Definitions differ across studies and authors, limiting consistency; therefore, the term is applied cautiously throughout the manuscript and explicitly defined when applied
COPC (chronic overlapping pain conditions) [2, 3]	A group of chronic pain disorders that frequently co-occur and share common underlying mechanisms, often involving central sensitization or nociplastic processes. Examples include fibromyalgia, irritable bowel syndrome, migraine, and temporomandibular disorder. These conditions are characterized by persistent pain, heightened sensitivity, and overlap in symptom profiles and comorbidities. The premise of this paper is that in some cases, COSP may fall under the broader umbrella of COPC

## Epidemiology

### Epidemiology of Chronic Overlapping Pain Conditions (COPCs)

Globally, COPCs are highly prevalent, with individual conditions affecting up to 1 in 7 people [13, 14], with similar patterns observed in the USA. In the USA, the three most prevalent conditions affecting both males and females are cLBP (~ 13.1%) [15], migraine (~ 11.7–14.7% for combined episodic and chronic cases) [16], and IBS (~ 6.1%) [17]. Across COPCs, female predominance and greater symptom severity in females are consistently observed (Table 2). Substantial overlap and co-occurrence further characterize COPCs, with headache disorders and FM frequently clustering with multiple conditions, suggesting shared vulnerability.

**Table 2:** Epidemiology of individual chronic overlapping pain conditions (COPC)

Condition	Prevalence	F:M ratio	Racial predilection in study	Age trend in study	Study/design
Chronic low back pain (cLBP)	13.1%	1.3:1	Whites	Peak age: 50–59 y	National Survey [15]
Migraine overall (chronic)	11.7–14.7% (0.91%)	3.1:1** (2.6:1)	Whites > Blacks	F peak age: 30–39 y M peak age 30–39 y	Population-based [21]
Irritable bowel syndrome (IBS)	6.1%	1.8:1**	NH† Whites	18–29 y < 30–59 y 18–29 y > 60+ y	National Survey [17]
Temporomandibular disorder (TMD)	4.6%	2.3:1**	NH Whites>NH Blacks	NH White F bimodal: 25–34 y and 45–54 y; Black F peak age: 55–64 y Black M peak age: 45–54 y	National Survey [35]
Chronic tension-type headache (cTTH)	2.2%	2:1	No predilection	No trend	Population-based [181]
Interstitial cystitis/bladder pain syndrome (IC/BPS)	0.8-2.7% (F) 0.3–1.2% (M)	2.3–2.7:1**	Variable findings	Variable findings	Population-based [182]
Fibromyalgia	1.8%	2.2:1**	Blacks=Whites Whites>Asians	F peak age: 60–69 y M peak age: 50–59 y	National Survey [33]
Myalgic encephalomyelitis/chronic fatigue syndrome (ME/CFS)	1.3%	1.9:1**	NH Whites	Peak age: 60–69 y	National Survey [34]
Vulvodynia*	8%	Female exclusive	White > Black > Hispanic	Limited	Population-based [36]
Endometriosis*	6.4%	Female exclusive	NH White > Hispanic>NH Black	No trend	National Survey [37]

*F* female, *M* male

^*^Female specific COPCs

^**^Estimated/calculated

^†^Non-Hispanic

Chronic low back pain is a leading cause of disability worldwide [18], with a point prevalence of 13.1% in the USA [15]. In 2020, more than half a billion individuals globally were living with low back pain, accounting for 7.7% of all years lived with disability [19]. In the 2019 National Health Interview Survey of 31,997 adults, 8.2% (2925 respondents) reported severe back pain (defined in the study as occurring on most or every day and rated as “a lot” over the prior three months) [20]; females comprised 58.4% of cases [20]. Prevalence was highest among adults aged 45–64 years (44.3%), followed by those aged ≥65 years (29.3%) and 18–44 years (26.4%) [20]. Whites had approximately 1.5 times higher odds of low back pain than Blacks (95% confidence interval [CI] 1.1–2.0) [15]. These findings underscore the disproportionate burden of cLBP among middle-aged adults, particularly females and Whites.

Migraine, encompassing both episodic and chronic forms, is similarly prevalent, with chronic migraine classified as a COPC. A systematic review of 26 publications from 11 US population-based studies reported an overall prevalence of 11.7–14.7%, with higher rates among females (17.1–19.2%) than males (5.6–7.2%) [16]. Chronic migraine prevalence was 0.91% among adults (1.3% for women and 0.5% for men) [16]. The American Migraine Prevalence and Prevention (AMPP) study of 162,576 individuals aged ≥12 years showed peak prevalence at ages 30–39 years in females (24.4%) and males (7.4%), with the lowest prevalence at ≥60 years (5.0% and 1.6%, respectively) [21]. Compared to Blacks, Whites had approximately 1.5–1.6 times higher odds, with similar estimates in both males and females [21]. Moderate or severe disability was reported by 22% [21], highlighting substantial functional burden in addition to disproportionate impact among females and midlife adults.

Irritable bowel syndrome demonstrates similar patterns. In a national survey of 88,607 adults aged ≥18 years, 5414 individuals (6.1%) met criteria [17]. All subtypes were more common in females, including IBS-C (constipation-predominant; odds ratio [OR] 1.7, 95% CI 1.6–1.9), IBS-D (diarrhea-predominant; OR 1.4, 95% CI 1.3–1.6), and IBS-M (mixed type; OR 2.1, 95% CI 1.9–2.3) [17]. Adults aged ≥60 years had lower odds compared with those aged 18–29 years (OR 0.8, 95% CI 0.6–0.95; 0.5, 95% CI 0.4–0.7; and 0.44, 95% CI 0.4–0.5, respectively) [17]. Similar to the other conditions, non-Hispanic Whites had higher odds of IBS compared to racial and ethnic minority groups [17]. Collectively, these findings further support shared features of female predominance, racial disparities, and greater burden in younger populations.

Although prevalence estimates are reported for individual conditions, comorbidity is common across COPCs. In the OPERA study of 655 adults examining the overlap among five COPCs (TMD, headache [TTH or migraine], IBS, cLBP, and FM), 32% had one COPC, 13% had two, and 8.3% had three or more [22]. Among individuals with “headache”, 51% had another COPC [22]. Overlap was especially frequent in FM, where 90% reported at least one additional COPC and 24% reported all five [22]. Similarly, 78% of those with TMD had another COPC, most commonly headache [22].

Overlap was also documented in the Multi-Site Clinical Assessment of ME/CFS, which included 923 participants (595 with ME/CFS and 328 healthy controls) from seven US specialty clinics [23]. Among those with ME/CFS, 76% had at least one COPC compared with 17.4% of healthy controls [23]. Within the ME/CFS group, chronic migraine or headache was most prevalent (48.1%), followed by FM (45.0%), cLBP (33.1%), and IBS (31.6%) [23], all more prevalent in females (Table 2). Compared to healthy controls, individuals with ME/CFS were nearly 150 times more likely to report FM, about 40 times more likely to report cLBP and about 14 times more likely to report IC/BPS [23], underscoring that overlap between ME/CFS and other COPCs is frequent, with females particularly affected.

Taken together, COPCs frequently occur in combination, and single-condition prevalence estimates likely underestimate true population burden. Accounting for overlap is essential to accurately quantify disease impact and to identify populations, particularly females, at highest risk.

### Epidemiology of Chronic Ocular Surface Pain (COSP)

The prevalence of COSP is not fully known but can be inferred from symptom-based surveys examining “dry eye disease (DED)” [9, 10]. Initially, symptoms such as “dryness”, “discomfort”, and “grittiness” were attributed to tear film abnormalities, thus these studies were referred to as DED epidemiological studies. However, increasing evidence demonstrates that ocular pain symptoms arise from multiple etiologies beyond tear dysfunction. Therefore, in the absence of an ocular surface examination, these frequency estimates more accurately reflect the prevalence of ocular surface pain (OSP) symptoms—or COSP when symptoms persist for more than three months [9].

For example, in the Blue Mountains Eye Study of 1174 residents of New South Wales, Australia aged ≥49 years (mean 60.8 years; range: 50–90; 55.8% females), 57.5% reported at least one OSP symptom in the past year (dryness, grittiness, itchiness, or discomfort) [24]. Itchiness was most often reported (~ 40%), followed by discomfort and grittiness (~ 30%), and dryness (~ 20%) [24]. Females more frequently reported OSP (age-adjusted OR 1.5, 95% CI 1.1–2.2), including dryness (OR 1.6, 95% CI 1.2–2.2) and discomfort (OR 1.6, 95% CI 1.2–2.1) [24]. Moderate to severe symptoms were reported in 10.6% of females and 6.0% of males (OR 1.6, 95% CI 1.1–2.2), with grittiness (OR 2.0, 95% CI 1.0–4.0) and discomfort (OR 1.7, 95% CI 1.1–2.7) more common in females [24], demonstrating both higher likelihood of OSP and greater severity in females.

Similar findings were noted in the Women’s Health Study, a study of 39,876 US female health professionals aged 45–84 years across 50 states [25]. Participants were asked how often their eyes felt dry or irritated (“constantly,” “often,” “sometimes,” or “never”) [25]. Dryness was reported sometimes by 28.3%, often by 5.7%, and constantly by 1.0% [25]. Similar frequencies were noted for irritation: 51.1%, 5.7%, and 0.6% [25]. Prevalence increased with age, from 5.7% among those aged <50 years to 9.8% among those aged ≥75 years. Racial differences were observed: compared with White females, Hispanic females (OR 1.8, 95% CI 1.2–2.8) and Asian females (OR 1.8, 95% CI 1.8–2.7) reported more severe symptoms [25], underscoring age and racial differences in OSP symptomatology. Similar influences were further supported by the Beaver Dam Eye Study, which examined 3722 predominantly White individuals in Wisconsin (mean age 65 years; range: 48–91 years; 57% females) [26]. Participants reported if they had experienced dry eyes (foreign-body sensation with itching, burning, or a sandy feeling, not related to allergy) for ≥3 months [26]. Overall, 14.4% responded ‘yes,’ and females had 1.29 times higher odds than males (95% CI 1.0–1.6) [26]. Prevalence increased with age (*p* < 0.001 for trend), rising from 8% in ages 48–59 years, to 15% in ages 60–69 years, and peaking at 20% in ages 70–79 years [26]. When considered concomitantly, both female gender and older age remained significant risk factors.

Global studies demonstrate similar patterns. In Bangkok, Thailand, 550 participants (mean 58.8 years; range 40–78; 72.5% females) completed a standardized questionnaire assessing dryness, foreign-body sensation, burning, and discomfort [27]. The most commonly reported descriptor was discomfort, with 20% endorsing it as occurring “often,” followed by burning (15%) and dryness (10%). In contrast, fewer participants reported discomfort or dryness “all the time” (2.8% for each symptom) [27]. Female gender was again identified as a risk factor [27].

In Singapore, 1004 participants (mean age 38.2 years; range 15–83 years; 55.9% females) were surveyed; 12.3% reported at least one symptom “often” or “constantly.” Dryness was most common (14.6% often, 9.7% constantly), followed by scratchiness (13.0% often, 7.8% constantly) and soreness (13.9% often, 5.9% constantly). Prevalence again increased with age, albeit non-significantly: compared with individuals aged < 25 years, the mid-age group had an OR of 1.3 (95% CI 0.7–2.2) and those aged > 45 years had an OR of 1.4 (95% CI 0.7–2.6) [28]. No significant ethnic differences were observed (Malays, Chinese, Indians, and others) [28], suggesting minimal ethnic variation and muted age trends in this population.

Extending beyond Asia, in Brazil, 3107 participants from five geopolitical regions (mean age 40.5 years; 65.6% females) completed the same questionnaire used in the Women’s Health Study [29]. Severe symptoms (“often” or “constantly”) were present in 4.9%, with female gender (OR 1.9, 95% CI 1.3–2.8) and age ≥ 60 years (OR 2.2, 95% CI 1.5–3.3) independently associated. Among females, dryness was reported often by 12.0% and constantly by 5.0% versus 5.0% and 1.0% in males; irritation was reported often by 17.0% and constantly by 6.0% versus 7.0% and 2.0%, respectively [29]. The semi-arid Northeast region had the highest frequency of severe symptoms (7.4%) [29]. These findings highlight female gender, older age, and environmental conditions as key influences on OSP prevalence.

Across studies, consistent epidemiologic patterns emerge (Table 3). Restricting estimates to moderate–severe or “often/always” symptoms, prevalence ranges from 5 to 34%, with a weighted female-to-male ratio of approximately 1.5–1.7:1 where stratified data are available. Females are consistently more affected, both in prevalence and in severity of reported symptoms. Age amplifies risk, particularly among females, with prevalence rising into the sixth and seventh decades, although some Asian cohorts show muted trends. Environmental conditions may also influence burden, as suggested by regional variation in Brazil.

**Table 3:** Summary of ocular surface pain (OSP) epidemiology by study

Study (year)	Symptom items	Frequency/severity**	F:M prevalence (%)	F:M OR (95% CI)	Race/ethnicity OR (95% CI)	Age trend OR (95% CI)
Blue Mountains (2003) [24]	Dryness, grittiness, itchiness, discomfort	Moderate–severe: 16.6%	10.6 vs 6.0	1.6 (1.1–2.2)	n/a	n/a
Women’s Health Study (2003) [25]	Dryness, irritation	Dryness often 5.7%, constant 1.0%	n/a	n/a	H vs W: 1.8 (1.2–2.8); A vs W: 1.8 (1.2–2.7)	≥75 y (9.8%) vs <50 y (5.7%)
Beaver Dam (2000) [26]	FBS, itching, burning	Overall “yes”: 14.4%	16.7 vs11.4	1.3 (1.0–1.6)	n/a	Peak 70–79 y (20%)
Bangkok Community (2006) [27]	Dryness, burning, FBS	Often: discomfort 21%, burning 15%, dryness 10%	39.1 vs 20.5	n/a	n/a	Peak 50–59 y (36.3%)
Singapore population (2015) [28]	Dryness, scratchiness	≥1 symptom often/constantly 12.3%	14.8 vs 9.0	0.8 (0.5–1.3)	n/a	No trend
Brazil National Survey (2018) [29]	Dryness, irritation	Severe symptoms 4.9%	n/a	1.9 (1.3–2.8)	n/a	≥60 vs <40 y: 2.2 (1.5–3.3)

*A* Asian, *CI* confidence interval*, FBS* foreign body sensation, *F:M* females:males, *H* Hispanic, *n/a* not available, *OR* odds ratio, *W* White

^**^Estimates are for moderate to severe OSP occurring often or all the time; however, symptom duration was not consistently reported, and as such it is unclear if OSP was chronic (≥3 months)

### Epidemiology of COSP within the COPC Framework

In the broader context of COPCs, COSP shares key features including female predominance and age-related increases. This pattern parallels conditions like cLBP, which is also more common in females and peaks in midlife, with prevalence comparable to COSP. As comorbidity is a defining feature in COPCs, the co-occurrence likely extends to COSP. Evidence supports this association: in one study comparing 31 participants with self-reported migraine, validated by questionnaire (13 meeting criteria for chronic migraine), to 219 controls without migraine, ocular pain intensity was significantly greater in both migraine groups compared with controls [30].

Similar associations have been observed with other COPCs. In a cohort of 154 South Florida Veterans, OSP was assessed with validated questionnaires (Neuropathic Pain Symptom Inventory applied to the Eye [NPSI-Eye; total score 0–10031] and a Numerical Rating Scale [NRS] of average and worst pain recall over 1 week, 0–10) and the presence of chronic pain conditions (lasting >3 months including headaches, complex regional pain syndrome, back pain, temporomandibular pain, and fibromyalgia) was captured. Two-step cluster analysis stratified patients into Low Pain (*n* = 57; chronic pain syndromes 2.5 ± 1.5; pain locations 1.1 ± 0.7) and High Pain (*n* = 97; chronic pain syndromes 6.2 ± 3.5; pain locations 3.8 ± 1.1) comorbidity groups. Compared with the Low Pain group, the High Pain group reported more severe OSP with neuropathic features (NPSI-Eye: 29 ± 23 vs 19 ± 19) and greater intensity (NRS: 4.5 ± 2.5 vs 2.9 ± 2.4). The High Pain group also reported higher non-ocular pain intensity (NRS: 6.0 ± 2.4 vs 3.9 ± 2.9), post-traumatic stress disorder (PTSD) (PTSD Checklist – Military Version: 45 ± 20 vs 36 ± 19; total: 17–85), and depressive symptoms (Patient Health Questionnaire 9 [PHQ9]: 10.8 ± 8.0 vs 6.9 ± 7.5; total: 0–27; all *p* < 0.05) [32]. This study highlights that greater COPC burden is associated with more severe OSP. Taken together, COSP shares key demographic features with COPCs, and shows a tendency to co-occur with COPCs and other chronic pain conditions, supporting consideration of COSP potentially as a distinct COPC.

## Risk Factors

### Risk Factors for COPCs

Risk factors for COPCs encompass demographic, environmental, lifestyle, psychosocial and genetic domains. Demographic patterns mirror epidemiologic trends, with advancing age [15, 17, 33, 34] and White race associated with greater risk across most conditions [15, 17, 33–37]. The most consistent demographic risk factor, however, is female gender, which is linked to higher disease burden across all 10 COPCs [15, 17, 33–37].

Environmental exposures and lifestyle behaviors contribute to several COPCs, including cLBP, migraine, and IBS. In cLBP, occupational factors are prominent. In a longitudinal survey of 3505 Finns aged ≥30 years, prolonged standing (>5 h/day), driving (>4 h/day), manual handling of weights >5 kg, and use of vibrating tools (>2 h/day) were associated with an increased risk of low back pain lasting >30 days compared with individuals without these exposures (OR 1.11–1.61) [38]. In migraine, environmental triggers such as weather, air travel, noise, and outdoor light exposure can precipitate attacks [39]. In a cross-sectional study of 1207 individuals with migraine (both episodic and chronic types), 75.9% reported identifiable precipitating factors for attacks, most commonly weather (53.2%), perfume or odor (43.7%), light exposure (38.1%), smoke (35.7%), and heat (30.3%) [40]. In IBS, smoking, air pollutants, pet ownership, and poor sanitation were associated with disease onset [41]. In a Sydney cohort of 767 participants, sharing a bedroom up to age 5 (OR 1.9, 95% CI 1.7–3.1, *p* = 0.01), exposure to herbivore pets (OR 1.7, 95% CI 1.1–2.5, *p* = 0.02), and poor hygiene (OR 4.4, 95% CI 1.9–10.2, *p* = 0.001) were associated with IBS [42]. Collectively, these findings highlight the contribution of environmental factors to the development of several COPCs.

Lifestyle factors further modulate risk. Overweight (OR 1.3, 95% CI 1.1–1.6) and obesity (OR 1.62, 95% CI 1.3–2.0) increase LBP risk compared to a normal BMI [38]. Sleep disturbance amplifies pain sensitivity: among 971 healthy adults, sleeping <6 hours or >9 hours was associated with greater next-day pain, with ≤3 hours increasing pain frequency by 81% relative to 6–9 hours [43]. Similarly, in a study of 53 rotating nurses, night-shift work increased sensitivity to electrically induced pain by 22.3% and heat pain by 26.5% (*p* < 0.001), underscoring the modulatory role of sleep in pain processing [44].

Beyond demographics and environment, psychosocial vulnerability—including negative affect, childhood trauma, low social support, and catastrophizing—plays a central role across COPCs; depression, anxiety, and distress can precede and predict pain onset [45, 46], and are robust predictors of transition to chronic pain, and impair analgesic response. In a double-blind, placebo-controlled crossover trial of 60 patients with discogenic LBP stratified by psychopathology (low, moderate, and high; 20 participants in each; based on Beck Depression Inventory II, the Pain Anxiety Symptoms Scale, and the NEO Personality Inventory–Short Form) intravenous morphine and placebo were administered on separate visits. Intravenous morphine produced 65.1% total pain relief in the low psychopathology group versus 41.0% in the high group (*p* = 0.03). Morphine-minus-placebo analgesia was 59.2% versus 21.7% (*p* = 0.0001), indicating diminished opioid response in those with higher psychopathology [47]. Similarly, distress also contributes to physical disability; in a meta-analysis of back and neck pain (*n* = 502), it explained 31% of variability in the pain–disability relationship [48].

Trauma exposure is another established risk factor. A meta-analysis demonstrated a 2.7-fold greater risk of FM, IBS, chronic fatigue syndrome, or TMD following trauma [49]. Strongest associations were observed for combat deployment (OR 3.1, 95% CI 1.7–5.5), PTSD (OR 2.9, 95% CI 2.4–3.6), emotional abuse (OR 2.1, 95% CI 1.6–2.8), and sexual abuse (OR 2.0, 95% CI 1.7–2.3) [49]. Similarly, in a longitudinal study of 7571 participants at age 7 and again at age 45, childhood hospitalizations (OR 1.7, 95% CI 1.3–2.4), financial crises (OR 1.6, 95% CI 1.3–1.9), and parental death (OR 2.0, 95% CI 1.1–3.7) were associated with increased risk of developing chronic widespread pain in adulthood, even after adjustment for adult psychological distress and social class [50].

Catastrophizing, a pain-specific construct involving helplessness, pessimism, rumination, and magnification of symptoms, independently predicts pain outcomes [51]. In a study of 655 adults examining five COPCs, catastrophizing was associated with TMD, headache, low back pain, and fibromyalgia (ORs = 1.4–1.8; all *p* < 0.01) [52]. Catastrophizing indeed correlates with depression, anxiety, distress, fear of pain, and anxiety sensitivity (*r =* 0.26–0.72) [53].

Genetic factors also influence pain facilitation and inhibition. Genes implicated in mood and stress regulation, including adrenergic receptor *β*, catechol-O-methyltransferase, dopamine receptor D4, μ-opioid receptor, and serotonin transporter affect pain processing [54]. Migraine has been linked to > 30 loci, notably methylenetetrahydrofolate reductase *(MTHFR)*, whose dysregulation may increase neuronal excitability via *N*-methyl-d-aspartate (NMDA) receptor activation [54]. Furthermore, transient receptor potential melastatin 8 (*TRPM8*), a cold-sensing receptor expressed on both peripheral sensory neurons and deep visceral afferents [55], has been identified across several genome-wide association studies (GWAS) as a migraine susceptibility gene [56, 57] and also implicated in IBS-C and IBS-M [58]. Interleukin (*IL)-1* and tumor necrosis factor (*TNF)-α* variants are linked to migraine susceptibility, generation, and clinical features [59–61]. Similarly, in cLBP, polymorphisms at the IL-1 gene locus have been associated with the pathogenesis of low back pain [62]. Additionally in IBS, polymorphisms in TNF superfamily member 15 (*TNFSF15*) have been associated with increased disease risk [63].

Finally, the presence of one COPC strongly predicts another, potentially reflecting a state of pain amplification [5], consistent with epidemiologic overlap as high as 90% in FM [22]. Together, these findings underscore the multifactorial and interrelated nature of COPCs across demographic, environmental, lifestyle, psychosocial, and genetic spheres.

### Risk Factors for COSP

Factors influencing COSP can be similarly categorized into demographic, environmental, psychological, lifestyle and genetic factors. Most risk factors are extrapolated from studies of “dry eye symptoms” or “DED,” given the substantial overlap in underlying mechanisms. Although the present focus emphasizes pain-related terminology, it is important to recognize this overlap when interpreting risk factors.

Demographic determinants parallel epidemiologic observations. Advancing age and female gender are consistently associated with increased risk for COSP across multiple studies [24, 26, 28, 29].

Environmental factors also contribute to OSP. Relative humidity (RH) has been implicated. In a Finnish crossover study of 290 office workers alternating between high humidity (30–40% RH) and “natural” low humidity (20–30%) for 3 weeks each, daily ocular pain scores (Likert 0–3) were significantly higher in the low-RH condition (0.39 vs 0.35; *p* < 0.05) [64]. Consistently, in a New Zealand study of 44 participants, increasing RH by 5.4% ± 5.0% using desktop humidifiers improved ocular comfort in 36% of participants compared with 5% without humidification (*p* < 0.001) [65].

Psychological wellbeing and sleep quality, which are intricately linked [66, 67], are also associated with ocular pain. In 141 South Florida Veterans (mean age 56 ± 5), validated questionnaires assessed sleep quality, mental health, and ocular pain severity. Linear regression demonstrated that ocular pain scores on NRS (across “now”, “average over 1 week” and “worst over 1 week”) were all significantly associated with the sleep disturbances question on the Pittsburgh Sleep Quality Index (PSQI) (*β* = 0.32–0.52, *p* < 0.0005). Numerical Rating Scale ocular pain “now” was also associated with depression severity on the Patient Health Questionnaire-9 (PHQ-9) (*β* = 0.28, *p* = 0.01). Similar relationships were noted for OSP measured on NPSI-Eye with PHQ-9 (*β* = 0.47, *p* < 0.0005) and sleep disturbances (*β* = 0.23, *p* = 0.02) [68], reinforcing relationships between ocular pain intensity, depression severity, and sleep disturbances.

Trauma exposure may confer additional risk. In an Italian study, investigators applied several questionnaires including the modified Self-Administered Leeds Assessment of Neuropathic Symptoms and Signs (S-LANSS) to 26 individuals with OSP, further subdivided into a centrally mediated group (S-LANSS ≥12, minimal surface findings, persistent pain after anesthetic, *n* = 9) and two peripherally mediated groups (S-LANSS ≥12; *n* = 10 and S-LANSS ≤12; *n* = 7) based on symptom burden. In the centrally mediated OSP group, pain severity strongly correlated with PTSD (R2 = 0.83) and depression (R2 = 0.93; both *p* < 0.05) scores, whereas relationships were weak in the peripheral groups (R2 = −0.13 to 0.25). Although limited by a small sample size, the findings raise the possibility that centrally mediated COSP is more closely linked to trauma and mood disorders [69].

As in COPCs, genetic factors likely contribute to ocular pain vulnerability, consistent with evidence that polymorphisms influence somatosensory processing and chronic pain risk [70]. Variants in *TRPM8* and *IL1* have been associated with OSP susceptibility in a Korean population [71] and separately, *TNF-α* polymorphisms with differential response to topical anti-inflammatory therapy [72].

Unique polymorphisms, independent of COPC-related variants, have also been implicated in OSP. In 389 veterans with burning and wind sensitivity quantified by NPSI-Eye, a GWAS identified one nucleotide polymorphism (SNP) reaching genome-wide significance (*p* < 5 × 10⁻⁸), along with 10 additional SNPs approaching significance (*p* < 5 × 10⁻⁷). The lead SNP was predicted to alter the binding motif of transcription factor Zbtb3, expressed in neural tissues, suggesting a potential pathogenic role in mediating OSP [73].

### Risk Factors for COSP within the COPC Framework

Taken together, COPCs and COSP share multiple overlapping risk factors. Psychosocial factors play a central role in both. Sleep disturbance, depression, anxiety, and catastrophizing are associated with greater pain severity [45, 46, 52, 68]. Similarly, a history of trauma likewise represents a shared vulnerability linked to persistent pain [49, 50, 69]. Environmental influences also overlap. In COPCs, triggers such as weather, light exposure, noise, and pollutants have been linked to symptom exacerbation [39–41], while in COSP, changes in temperature and humidity are associated with increased ocular pain and discomfort [64, 65]. Genetic contributions also appear relevant across both conditions [59–61, 71], with evidence of shared genetic susceptibility. In a large twin study using the TwinsUK database, OSP captured under the diagnosis of dry eye (a terminology limitation, as pain-specific phenotyping was not performed) clustered with chronic widespread pain and vulvodynia, with two shared latent genetic factors and an overall heritability of approximately 66% [74]. These findings suggest that OSP may share elements of a common genetic architecture with certain COPCs.

## Pathophysiology

### Pathophysiology of COPCs

In a subset of individuals with COPCs, amplified and/or dysregulated neural signaling and sensory processing within the CNS (i.e., nociplastic pain) plays a pathogenic role in the onset and maintenance of chronic pain [75]. Nociplastic pain is conceptualized as a quantitative mechanistic descriptor that occurs on a continuum, rather than a binary state across COPCs [12]. Clinically and neurobiologically, it is characterized by pain disproportionate to objective findings (symptom–sign discordance), multisite or widespread pain, and non-pain CNS-mediated symptoms including fatigue, sleep disturbance, mood disorders, and cognitive impairment [12, 75]. Individuals with nociplastic pain typically exhibit multisite and localized hyperalgesia and allodynia to somatosensory stimulation (e.g., pressure, heat) and increased sensitivity to environmental stimuli (e.g., light, sound), which is conceptualized as “multisensory hypersensitivity" more broadly [75]. In subgroups of nearly every COPC, these nociplastic features have been identified either in isolation, as in fibromyalgia, a COPC and prototypical nociplastic pain condition, or as part of a heterogeneous mixed-pain state with ongoing nociceptive pain (which arises from actual or threatened damage to non-neural tissue and is due to the activation of nociceptors [76]) or neuropathic pain (caused by a lesion or disease of the somatosensory nervous system [76]).

Neuroimaging studies demonstrate altered functional connectivity among the default mode (DMN), salience (SLN), and sensorimotor (SMN) networks in fibromyalgia and other COPCs, reflecting a shared neurobiological signature of central sensitization [77]. The DMN supports self-referential processing and pain modulation, with altered activity associated with depression, anxiety, sleep disturbance, and impaired decision making [78, 79]. The SLN mediates detection of aversive stimuli and affective pain processing, with dysfunction linked to maladaptive cognitive and emotional responses across chronic pain conditions [79, 80]. The SMN network integrates sensory input and motor output, and altered connectivity has been associated with abnormal somatosensory processing in chronic pain states [77, 81].

Increased DMN–SLN–SMN connectivity has been reported at rest and during sensory stimulation across IC/BPS, cLBP, and migraine [77]. In chronic pelvic pain, individuals with comorbid IC/BPS, widespread pain or FM exhibited elevated SLN–SMN connectivity, whereas those with isolated pelvic pain showed connectivity patterns more similar to those of healthy controls, suggesting altered network coupling as a potential mechanism underlying widespread pain perception [82].

Altered network connectivity in COPCs may reflect imbalances in neurotransmission, including increased excitatory signaling and/or reduced inhibitory signaling [77]. Individuals with FM demonstrate increased excitatory neurotransmission within the SLN [83] and DMN [84], with elevated glutamate and glutamine levels measured by magnetic resonance spectroscopy. Higher concentrations, particularly within the insula and posterior cingulate cortex, correlate with greater clinical pain severity in FM [84, 85]. Altered inhibitory neurotransmission has also been reported, including reduced γ aminobutyric acid (GABA) levels in SLN regions [86, 87] and increased GABA levels within the DMN [88] across various COPCs.

Consistent with these findings, quantitative sensory testing (QST) demonstrates heightened sensitivity to both painful and normally non-painful stimuli in individuals with nociplastic pain features compared with pain-free individuals and those with chronic pain but without nociplastic features [89, 90].

Importantly, nociplastic vulnerability may emerge early in life. In a 2022 longitudinal study of pain-free children in the Adolescent Brain Cognitive Development Study, those who developed multisite pain 1 year after neuroimaging showed increased baseline SLN–SMN–DMN connectivity compared with those who remained pain-free [91]. No significant baseline differences in cortical thickness or gray matter volume were observed in children who developed multisite pain, suggesting that functional alterations precede pain onset, whereas structural changes may occur as a consequence rather than a cause [91, 92]. These findings suggest that alterations in large-scale brain network connectivity, potentially emerging early in life, may represent an underlying vulnerability to chronic pain development.

**Fig. 2:**
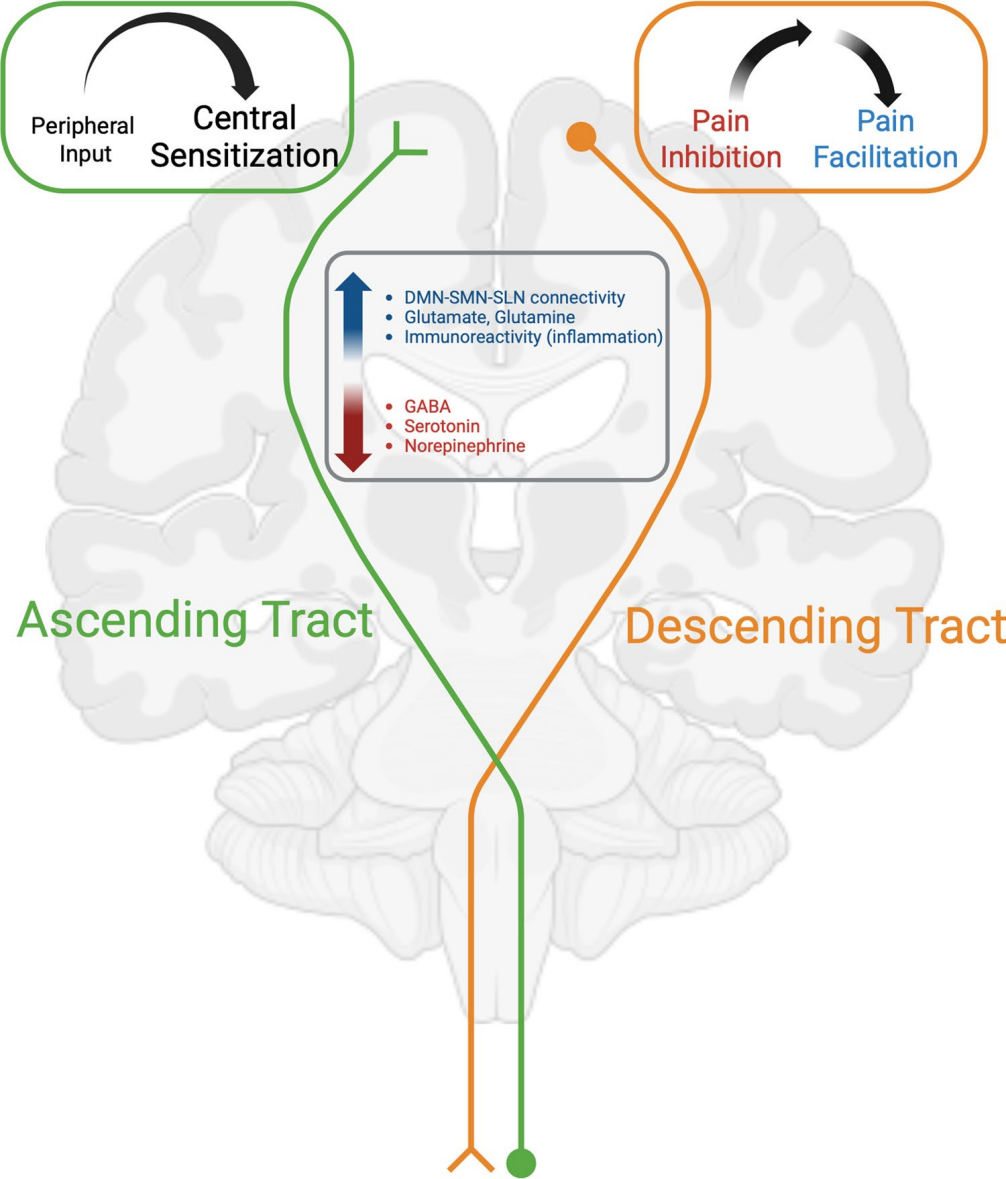
Definition and pathophysiology of nociplastic pain. Nociplastic pain is defined as pain arising from altered nociceptive processing within the central nervous system rather than from identifiable tissue injury or nerve damage. Current understanding suggests that nociplastic pain is driven by abnormal neural connectivity and heightened pain sensitivity through multiple mechanisms that include, but are not limited to: (i) increased peripheral input that sustains central sensitization; (ii) increased default mode–salience–sensorimotor network (DMN–SLN–SMN) connectivity, reflecting central sensitization; (iii) dysfunction of descending pain modulatory pathways, with a shift from inhibitory to facilitatory signaling; (iv) alterations in neurochemistry (e.g., reduced inhibitory γ-aminobutyric acid [GABA], serotonin, and norepinephrine, and increased excitatory glutamate and glutamine); and (v) neuroinflammation and other concurrent risk factors (not shown) including genetic, epigenetic, hormonal, and psychosocial factors. Our premise is that, in some cases, COSP is driven predominantly by nociplastic mechanisms, as outlined above, rather than by ongoing nociceptive input from tear film or ocular surface abnormalities

Another mechanism involves dysfunction in descending and ascending pain modulatory systems. Reduced inhibitory control within the periaqueductal gray–rostral ventromedial medulla (PAG–RVM) circuit can shift descending modulation toward facilitation, amplifying pain signaling [93]. In the ascending tract, persistent peripheral input can similarly promote pain facilitation (Fig. 2) via synaptic growth and reorganization, thereby leading to altered central processing (i.e., neuroplasticity) [93].

Overall, nociplastic pain may arise through bottom-up processes driven by sustained peripheral input or top-down dysregulation of descending modulation [93]. Genetic, epigenetic, inflammatory, hormonal, environmental, and psychosocial factors may individually or collectively contribute [75]. Together, these mechanisms yield heightened neural gain and sensory amplification, producing pain that is real but disproportionate to peripheral pathology.

### Pathophysiology of COSP

The cornea is richly innervated by the ophthalmic branch (V1) of the trigeminal nerve [94, 95], containing polymodal nociceptors responsive to mechanical, thermal, and chemical, and inflammatory stimuli [96–98]. While many cases of OSP stem from tissue damage or inflammation, a subset of patients experience persistent pain despite minimal or absent visible pathology, paralleling subsets of COPCs. Nociceptive ocular pain typically arises from tear-film instability, epithelial injury, or ocular surface inflammation [99, 100], whereas neuropathic and nociplastic forms involve maladaptive neural processing.

Neuropathic pain results from recognized nerve injury or disease, often peripheral, such as after surgery, infection, or in systemic conditions that damage nerve (e.g., Sjögrens disease, diabetes) [101]. Tissue damage and inflammation of the ocular surface can lead to peripheral axonal injury and the release of pro-inflammatory mediators, resulting in increased sensitivity of peripheral nerves, amplification of peripheral pain signaling, and sensory abnormalities [102, 103]. As it relates to the ocular surface, this condition is referred to as neuropathic corneal pain (NCP). Over time, persistent peripheral input may drive central sensitization, characterized by heightened responsiveness of central neurons to similar magnitudes of input and increased pain perception [102–104].

Within this continuum, and extending beyond purely neuropathic mechanisms, increasing evidence indicates that some patients with COSP exhibit nociplastic features, including symptom-sign discordance (i.e., greater pain than ocular surface signs). Even when ocular surface abnormalities are present (e.g., tear instability, reduced tear production, epithelial disruption, or inflammation), these findings may correlate poorly with reported pain severity [105, 106].

Consistent with COPCs, a subset of COSP patients demonstrate multisite and regional hyperalgesia, impaired pain inhibition (Fig. 2), and prolonged after-sensations on QST, indicating amplified central processing [106–108]. For example, in a study of 118 South Florida Veterans (mean age 60 years; 89% male), hot pain temporal summation (HPTS) at the forearm, a marker of central sensitization, was associated with greater OSP. HPTS and anxiety explained 17% of the variance in ocular burning intensity (*r =* 0.41; *p* < 0.001). Hot pain temporal summation, PTSD symptom severity, and tear breakup time together explained 25% of variance in wind sensitivity (*r =* 0.50; *p* < 0.001) and 30% of variance in total NPSI-Eye scores (*r =* 0.55; *p* < 0.001) [108], indicating heightened sensitivity from enhanced central excitability at sites remote from the eye in a subset of OSP patients.

Similarly, in a study of 326 South Florida Veterans (mean age 62 years; 92% men), prolonged aftersensations to noxious hot and cold stimuli at periocular and remote sites correlated with symptoms-sign discordance scores (derived from questionnaire responses and ocular examination findings; range −1 to 1, with positive values reflecting greater symptoms than signs). Aftersensations and temporal summation at the forehead and forearm were positively associated with discordance, including HPTS at the forehead (*r =* 0.24; *p* = 0.003) and forearm (*r =* 0.17; *p* = 0.03), and cold pain temporal summation at the forehead (*r =* 0.19; *p* = 0.01) and forearm (*r =* 0.16; *p* = 0.04)106. These findings support a role for impaired descending pain inhibition and central pain amplification in a subset of OSP patients.

Additional evidence comes from a cross-sectional study of 224 South Florida Veterans (mean age 62 years; 91% male) assessing responses to topical anesthetic [109]. Individuals with persistent pain after anesthetic application reported higher ocular pain intensity despite similar ocular surface signs compared with those whose pain fully resolved. Furthermore, the persistent pain group demonstrated increased cutaneous pain sensitivity at sites remote from the eye [109], consistent with nociplastic pain mechanisms.

Functional magnetic resonance imaging (fMRI) studies further support a link between COSP and nociplastic pain, at least in some individuals. In a study of 16 US Veterans (8 with COSP and light sensitivity and 8 controls), individuals with COSP showed increased light-evoked activation in S1 (part of SMN [110]), insular, and anterior mid-cingulate cortex cortices (both part of SLN [111]), regions implicated in sensory and affective pain processing [112]. These findings parallel altered connectivity of DMN-SMN-SLN network reported in COPCs.

In a complementary study by the same group (37 with COSP; 16 controls without OSP), participants with COSP demonstrated increased functional connectivity between the trigeminal nucleus and accumbens, and among the putamen, left caudate, and dentate gyrus subregion of the thalamus [113]. Although these regions are not cortical nodes of DMN-SMN-SLN, they function as subcortical and brainstem hubs integrating nociceptive, affective, and sensorimotor signals and may exhibit abnormal coupling with cortical pain networks in chronic pain states [114–118]. Together, these studies demonstrate altered light-evoked brain activation and differences in functional connectivity within cortical and subcortical pain-related networks in individuals with COSP compared with controls, patterns that resemble those reported in COPCs.

### Pathophysiologic Mechanisms for COSP within the COPC Framework

Chronic ocular surface pain lies along a pain continuum encompassing nociceptive, neuropathic, and nociplastic features, analogous to patterns observed in some COPCs. Evidence of peripheral nerve alterations, alongside CNS changes and altered cortical processing in subsets of patients, suggests overlapping mechanisms between COSP and other chronic pain states. Viewing COSP within a nociplastic framework shifts the focus from a purely localized ophthalmic disorder toward a broader, system-level pain phenotype. Consistent patterns across anesthetic response, QST indices, and neuroimaging findings, similar to those reported in COPCs, support shared nociplastic pathways in at least a subset of individuals with COSP.

## Diagnosis

### Diagnosis of COPCs

Diagnosis of individual COPCs relies on condition-specific criteria supported by clinical assessment, detailed history, and validated questionnaires rather than definitive biomarkers [119]. Clinicians document pain location, severity, chronicity, temporality, characteristics and widespread distribution using a body map [119], while assessing psychological comorbidities such as depression, anxiety, fatigue, sleep disturbances, and cognitive changes, all of which may support a nociplastic mechanism. Historical features such as family or childhood pain history [119] and prior trauma [49] can further suggest nociplastic vulnerability.

Several validated instruments assist evaluation. The Fibromyalgia Survey Criteria provide a composite symptom severity score capturing widespread pain, fatigue, cognitive symptoms, and sleep quality [120]. Comorbid symptoms can be further assessed using the National Institutes of Health Patient-Reported Outcomes Measurement Information System (PROMIS) [121]. The Central Sensitization Inventory can further quantify symptom burden related to central sensitization in relation to nociplasticity [122]. Additional tools include the Patient Health Questionnaire-4 for depression and anxiety [123], the Pittsburgh Sleep Quality Index for sleep disturbances [124], and the Multidimensional Fatigue Inventory for fatigue [125].

Condition-specific diagnostic criteria are available: the Analgesic, Anesthetic, and Addiction Clinical Trial Translations Innovations Opportunities and Networks–American Pain Society Pain Taxonomy (AAPT) 2019 [126] and the American College of Rheumatology (ACR) 2016 criteria for FM [120]; Rome IV for IBS [127]; the International Classification of Headache Disorders 3rd Edition (ICHD-3) for migraine [128]; and the Diagnostic Criteria for Temporomandibular Disorders (DC/TMD) criteria for TMD [129]. Clinicians must also remain alert for red-flag features that may indicate structural, infectious, inflammatory, or malignant disease, particularly in cLBP [130], IBS [131], and chronic migraine [132].

Because COPCs frequently overlap, screening across conditions can be challenging. To address this, Schrepf and colleagues developed a universal COPC Screener (COPC-S) through expert consensus and patient input. Thirty experts refined electronic self-report criteria for the 10 COPCs. In a counter-balanced study of 30 patients, the COPC-S showed high agreement with physician criteria (*κ =* 0.813, *p* < 0.001) and was rated clear and easy to use. Mean completion time was approximately 10 minutes (range 3–20 minutes) [3]. Although tested in a small, predominantly White sample (83%), these findings suggest the COPC-S may serve as a promising cross-condition screening tool.

### Diagnosis of COSP

Diagnosing chronic ocular surface pain (COSP) begins with careful history-taking, incorporating validated questionnaires to capture ocular symptoms, and non-ocular symptoms burden (mental health, sleep, quality of life) [133–135], alongside slit-lamp examination. This combined approach helps suggest a predominant pain mechanism, either nociceptive, neuropathic, or nociplastic, while recognizing that many patients exhibit mixed phenotypes [10]. For example, some symptoms, such as grittiness, are more likely to be nociceptive [136, 137], whereas others, such as burning, sharp or shooting pain, and sensitivity to wind and light, may suggest a neuropathic or nociplastic component (Table 4). Symptoms beyond the ocular surface may also provide clues to pain type; in NCP and nociplastic subtypes, greater symptom severity (e.g., burning, evoked pain provoked by wind, light, or air temperature) has been associated with poorer mental health, sleep quality, and quality of life [12, 134].

**Table 4:** Common contributors and symptoms across chronic ocular surface pain (COSP) phenotypes

Phenotype	Frequent symptoms	Common contributors
Nociceptive	Grittiness/foreign body sensation, irritation [136, 137]	Periocular/adnexal conditions: ▪ Periocular dermatologic disease (e.g., rosacea, seborrheic dermatitis [139]) ▪ Eyelid abnormalities (e.g., entropion, ectropion [139], lid laxity, ptosis [140]) Tear film/Meibomian dysfunction: ▪ Meibomian gland dysfunction (e.g., abnormal meibum quality [141]) ▪ Tear instability or reduced tear production [142] Ocular surface integrity/inflammation: ▪ Epithelial disruption [142] ▪ Ocular surface inflammation [143] Iatrogenic: ▪ Ocular medications [183, 184]
Neuropathic	Burning, pins and needles, sharp shooting, pressure-like pain, cold/wind sensitivity, abnormal corneal sensation [185, 186]	Direct peripheral nerve injury: ▪ Trauma [187] ▪ Toxic chemical exposure [188] Central nervous system damage: ▪ Stroke [189, 190] ▪ Demyelinating disorders/lesions [191] Systemic diseases: ▪ Small-fiber polyneuropathy [187] ▪ Fibromyalgia [187] ▪ Autoimmune conditions (e.g., Sjögren’s disease, lupus, sarcoidosis, inflammatory bowel disease, celiac disease [187]) ▪ Diabetes mellitus [187, 192] Iatrogenic: ▪ Refractive surgery [193, 194] ▪ Cataract surgery [195] ▪ Radiation keratopathy [101]
Nociplastic	Similar to neuropathic phenotype, often with multisite pain, light sensitivity*, generalized sensitivities (sound, odor), local and remote allodynia, and hyperalgesia* [119, 196]	Bottom-up factors: ▪ Sex hormones [197, 198] ▪ Dysautonomia [199, 200] ▪ Dysregulated immune system [201] ▪ Ongoing nociceptive input [77, 93] Top-down factors: ▪ Shared genetic/epigenetic components [12, 74] ▪ Psychological comorbidities (trauma, depression, anxiety, post-traumatic stress disorders [46, 52])

Other contributors to all phenotypes include: environmental factors [64, 65], sleep quality [68], lifestyle factors [162, 163] and genetic influences [59–61, 71]. Furthermore, patients may have more than one contributor to their ocular pain without a single predominant phenotype

^*^More common in the central neuropathic or nociplastic phenotype than in the peripheral neuropathic phenotype but can occur in both

After clinicians document pain characteristics, relevant comorbidities are important to elicit, including previous ocular surgery or trauma, autoimmune disease, systemic chronic pain conditions, sleep apnea, thyroid abnormalities, and ocular and systemic medication use [138], all of which can inform phenotypic stratification.

Examination proceeds from the periocular structures to the ocular surface. Periocular dermatological conditions (e.g., rosacea, seborrheic dermatitis [139]), eyelid abnormalities (e.g., entropion, ectropion [139], lid laxity, or ptosis [140]), meibomian gland issues (e.g., abnormal meibum quality or anatomy [141]), tear instability (e.g., low tear break up time), insufficient tear production and volume, epithelial disruption [142], and ocular surface inflammation [143] may underline nociceptive pain.

Equally important is assessing nerve function, as dysfunction may indicate neuropathic or nociplastic mechanisms. Corneal sensation can be evaluated qualitatively (cotton wisp) or quantitatively (Cochet Bonnet or Brill aesthesiometer) [144]. Both reduced and heightened sensitivity have been described in peripheral neuropathic pain, whereas nociplastic pain more often presents with normal or heightened sensitivity [138]. Facial sensitivity should also be assessed, as reduced or heightened sensitivity may indicate trigeminal neuropathy; sensory loss involving one or more divisions of the trigeminal nerve may reflect underlying structural or systemic pathology and warrants further evaluation [145]. In either case, alterations in sensation beyond the eye further support that the pain etiology is not confined to the ocular surface. In vivo confocal microscopy (IVCM), which provides qualitative imaging of the subbasal nerve plexus, may also be useful, as some individuals with peripheral neuropathic pain demonstrate decreased basal nerve density and structural abnormalities such as micro-neuromas [146]. However, IVCM currently lacks standardized quantitative metrics, and similar nerve alterations have been reported in systemic pain conditions; moreover, CNS injury may secondarily affect peripheral nerves. Thus, further data are needed to guide interpretation and integration of IVCM findings in clinical assessment.

Signs suggestive of a central nociplastic component to COSP include a positive anesthetic challenge, in which pain persists after topical anesthetic application (non-ocular surface source of pain [109]) and excessive light sensitivity in an otherwise normal eye (termed photophobia or photo-allodynia when painful [112, 147]). These features are incorporated into validated instruments such as the NPSI-Eye [31] and Visual Light Sensitivity Questionnaire 8 (VLSQ-8) [148] to aid phenotypic classification.

### Diagnosis of COSP within the COPC Framework

Although COPCs and COSP involve different organ systems, their diagnostic approaches share core principles. Both rely on careful clinical assessment, detailed history, and validated questionnaires to characterize pain and its functional impact, including mood disturbance, fatigue, sleep disruption, and cognitive symptoms. Trauma and family history of chronic pain are relevant in both settings.

Chronic ocular surface pain intersects with the COPC framework when nociplastic mechanisms are suspected, particularly in individuals with coexisting widespread pain, prior COPCs, or associated non-pain conditions such as insomnia or mood disorders. Features suggestive of nociplastic COSP include pain disproportionate to ocular findings, light-evoked pain in a normal eye, persistence after topical anesthetic, and pain extending beyond the ocular surface [12, 69].

In both COSP and COPCs, clinicians must determine whether nociplastic mechanisms contribute to the phenotype, often alongside nociceptive and/or peripheral neuropathic factors. Accurate phenotyping is essential for mechanism-based management and optimized treatment of systemic and ocular chronic pain conditions.

## Treatment

### Treatment Considerations for COPCs with a Nociplastic Pain Component

Management of nociplastic pain typically involves multidisciplinary, multimodal strategies targeting central mechanisms while addressing comorbidities that may amplify pain. Establishing realistic expectations and providing structured education should precede a stepwise plan, such as that proposed by the US Veterans Administration [149], beginning with nonpharmacologic measures and escalating to pharmacologic therapy based on symptom severity [119, 150].

Early care should follow a patient-centered biopsychosocial model emphasizing lifestyle modifications such as weight management, physical activity, sleep hygiene, and stress reduction, while fostering an internal locus of control and self-management [119].

Nonpharmacologic interventions are foundational, particularly for comorbidities, and include cognitive behavioral therapy to address catastrophizing, as well as mindfulness, acceptance-based strategies, psychodynamic therapy, biofeedback, and hypnotherapy [119, 150].

Pharmacologic agents are adjunctive when nonpharmacologic strategies are insufficient or symptoms remain severe. Traditional analgesics, including nonsteroidal anti-inflammatory agents, acetaminophen, and opioids are generally ineffective in predominantly nociplastic pain phenotypes. In fact, individuals with nociplastic pain tend to respond poorly and adversely to opioids due to elevated endogenous opioids, opioid-induced hyperalgesia, and sleep disruption [119, 150].

In contrast, low dose naltrexone (LDN), an opioid antagonist, has demonstrated efficacy through paradoxical analgesic and anti-inflammatory systemic effects. A systematic review of nine studies evaluating LDN in FM proposed two mechanisms of action: 1) enhancement of endogenous endorphin function and 2) neuroprotective and anti-inflammatory effects by suppressing microglial activation [151].

Other strategies include neuromodulatory agents. A meta-analysis of 185 randomized controlled trials (221 publications; 5 systematic reviews) across chronic pain conditions, including FM, cLBP, and chronic headaches, showed modest short-term improvements of 5–20 points (0–100 scale) in pain and function with alpha 2 delta (α2δ) ligands and serotonin-norepinephrine reuptake inhibitors (SNRI) over 1–6 months; longer-term outcomes were infrequently evaluated [152]. Of note, centrally acting non-opioid analgesics such as tricyclic antidepressants (TCA), SNRIs, and α2δ ligands may cause fatigue and cognitive side effects [152].

Nonpharmacologic neuromodulation strategies have also been explored [153]. In a masked, randomized, placebo-controlled trial of 41 individuals with FM, noninvasive transcutaneous electrical nerve stimulation (TENS; 100 Hz, 200 μs at maximal tolerable intensity applied at either cervical thoracic junction or lumbar-sacral junction by preference) improved movement-related pain and fatigue by approximately 20% (on a 0–10 scale, compared to resting state, *p* < 0.05) compared with sham and no TENS [154]. While the mechanisms underlying the benefits of TENS remain unclear, proposed mechanisms include 1) inhibition of peripheral nociceptors and ascending central pathways and/or 2) modulation of descending pain pathways [155, 156]. Repetitive transcranial magnetic stimulation (rTMS) targeting the primary motor cortex has also reduced pain intensity and improved quality of life in FM [157–159].

Lastly, some condition-specific therapy is also available. For example, botulinum toxin type A (BoNT-A) for chronic migraine is thought to reduce pain by inhibiting the release of calcitonin gene-related peptide and other nociceptive neuropeptides involved in peripheral sensitization of dural nociceptors [160].

Given clinical heterogeneity, treatment should align with the suspected pain mechanism. Therapies effective across multiple COPCs may be appropriate in selected patients, particularly given frequent overlap among these conditions.

### Treatment for COSP

Management of COSP follows a stepwise, multimodal approach addressing nociceptive, neuropathic, and nociplastic contributors, with treatment individualized to the patient’s pain phenotype [10]. In nociplastic presentations, where symptoms-sign discordance is common, management should also address comorbidities, similar to COPC care.

As with COPCs, patient education should precede treatment. Because the ocular surface can be disrupted by external factors, modifiable environmental contributors such as temperature [161], humidity [65] and prolonged screen time [162], and poorly fitted CPAP with air leakage [163] should be optimized. Addressing comorbidities that amplify pain may require interdisciplinary involvement, including behavioral health [164].

Regardless of phenotype, treatment begins with optimization of ocular surface health. In individuals with autoimmune-mediated inflammation, chronic anti-inflammatory therapies such as topical cyclosporine or lifitegrast may be beneficial [138]. When nociceptive input arises from meibomian gland dysfunction, options include lid hygiene, oral azithromycin or doxycycline, thermal pulsation [165], and correction of anatomic abnormalities, as indicated [138], although these have not been formally evaluated in randomized trials for COSP.

If pain persists despite adequate control of nociceptive sources, and neuropathic or nociplastic mechanisms are suspected—such as burning pain, wind or light sensitivity, extraocular pain, or psychological comorbidities—neuromodulatory strategies should be considered. Autologous blood-derived topical products are frequently used for peripheral neuropathic components [166]. Less data are available for topical insulin [167], recombinant human nerve growth factor [168], or TRPM8 agonists [169] in COSP, yet these products are available (commercially or through compounding) and commonly used for this purpose.

When a central component is suspected, aforementioned topical neuromodulation alone is often insufficient. Oral neuromodulators such as gabapentin or pregabalin [170] and TCAs [171] may be used, often combined with duloxetine [172] or LDN [173]. Because TCAs have anticholinergic effects, caution is warranted in individuals with low tear production [138, 171].

Adjuvant agents may benefit selected patients with COSP. In individuals with prominent light sensitivity or headache history, treatments used for chronic migraine, including TENS/trigeminal nerve stimulation (TNS) or BoNT-A, may offer benefit [174]. In 18 South Florida Veterans with OSP who used TNS for 6 months, ocular pain decreased by 31.0% and photo-allodynia by 36.0% compared with baseline (*p* < 0.01) [175]. Similar benefit has been reported with BoNT-A. In a study of 27 individuals with neuropathic or nociplastic features (mean age 52.6 years; 55.6% female; 74% with migraine), BoNT-A injections (35 units, increased to 100 units in selected patients) improved ocular pain at 1-month in 20 participants, with 25% reporting mild, 45% moderate, and 30% marked benefit. Improvements were also observed in light sensitivity (37%), wind/air sensitivity (33%), and quality of life (59%) [176]. Collectively, these findings suggest that BoNT-A may provide symptomatic relief for ocular pain and associated neuropathic features.

In a separate study, a modified BoNT-A protocol was applied to individuals with COSP but without a migraine history. Four participants who received a single 35-unit session across seven forehead sites reported decreased photophobia (4.8 ± 0.4 to 3.25 ± 0.4) and ocular discomfort (4.5 ± 0.6 to 2.25 ± 1.0) at 1-month follow-up [177], demonstrating potential efficacy of BoNT-A in patients with COSP but without migraine.

Periocular nerve blocks targeting supraorbital, supratrochlear, infratrochlear, and infraorbital nerves with long-acting anesthetic combined with corticosteroid [170] may also be used, particularly in individuals with cutaneous allodynia [11]. Other approaches warranting further investigation but potentially considered in select cases include autonomic ganglion blockade (e.g., sphenopalatine or stellate [10]) and acupuncture [101, 178].

### Treatment of COSP within the COPC Framework

Overall, key treatment approaches converge between COPCs and a subset of COSP, as both emphasize patient education and interdisciplinary care to address comorbidities. Pharmacologic overlap is notable, particularly with therapies targeting maladaptive central pain processing, and favorable responses to neuromodulatory treatments support shared central mechanisms (i.e., overlap with chronic migraine management and treatment). Some divergence is also evident, particularly because COSP may present with identifiable tissue damage (as do some COPCs such as cLBP with a predominant nociceptive component [179]), thereby necessitating nociceptive targeted therapy, such as with anti-inflammatory therapy in appropriate cases. Chronic ocular surface pain also involves a broad range of topical treatment options, whereas systemic medications are more commonly used in COPCs.

These similarities and distinctions highlight the importance of identifying the dominant pain phenotype and overlapping conditions to guide therapy. In nociplastic COPCs, addressing comorbidities is essential for effective management [164]. In COSP, where nociceptive, neuropathic, and nociplastic mechanisms often coexist, multimodal and appropriate interdisciplinary approaches remain essential to achieving optimal outcomes.

### Limitations and Knowledge Gaps

Several important limitations warrant consideration when interpreting the current literature on COSP and its relationship to COPCs. First, for COSP, much of the evidence derives from studies conducted under the broad and imprecise label of DED. Historically, ocular pain symptoms were frequently subsumed within DED cohorts without systematic phenotyping of pain duration, quality, or underlying mechanisms. As a result, many prior studies likely included heterogeneous populations in which nociceptive, neuropathic, and nociplastic contributors were not distinguished, complicating efforts to isolate findings specific to COSP.

Consequently, insights into COSP must often be inferred by re-examining older studies through a contemporary pain framework, rather than derived from investigations explicitly designed to study COSP. This approach introduces uncertainty and highlights the need for prospective studies that specifically define COSP, apply standardized chronicity criteria, and incorporate mechanistic subtyping using clinical features, QST, and neurobiological measures.

Second, although COPCs provide a useful conceptual framework, they encompass a diverse group of conditions with varying degrees of overlap in clinical presentation, comorbidities, and underlying mechanisms.

Future research should focus on rigorously phenotyped COSP cohorts and examine condition-specific overlaps with individual COPCs, rather than assuming uniform similarity across the entire COPC spectrum. Such work will be essential to clarify shared and distinct mechanisms and to guide mechanism-based diagnosis and management.

## Conclusion

Despite these limitations, current evidence underscores the multidimensional nature of COSP and its substantial overlap with COPCs across epidemiology, risk factors, pathophysiology, diagnosis, and treatment. Chronic ocular surface pain frequently co-occurs with other COPCs, with epidemiologic and genetic studies suggesting shared susceptibility. In addition, a subset of patients with COSP exhibits clinical and neurobiological features consistent with nociplastic pain, including symptom-sign discordance, persistent pain despite topical anesthesia or surface-directed therapy, multisite pain, and remote hypersensitivity. Similar risk profiles, psychosocial comorbidities, chronic symptom burden, and response to centrally targeted therapies further support a shared CNS-driven mechanism linking COSP and COPCs.

Clinically, this overlap underscores the importance of mechanism-based assessment and screening for coexisting conditions. Such an approach guides treatment selection, minimizes ineffective interventions, and facilitates coordinated interdisciplinary care.

Overall, advancing our understanding of the shared and divergent mechanisms underlying COPCs and COSP is foundational to future diagnostic and therapeutic progress. As accumulating evidence increasingly places COSP within a similar mechanistic framework of COPCs, we propose that, in some individuals, COSP may represent a COPC. More broadly, given the substantial overlap in clinical features and underlying mechanisms, COSP may warrant conceptual consideration as an additional member of the COPC cluster.
